# Dysfunctional Senescent Herpes Simplex Virus-Specific CD57^+^CD8^+^ T Cells Are Associated with Symptomatic Recurrent Ocular Herpes in Humans

**DOI:** 10.3390/v17050606

**Published:** 2025-04-24

**Authors:** Aziz A. Chentoufi, Arif A. Khan, Ruchi Srivastava, Sweta Karan, Yassir Lekbach, Hawa Vahed, Lbachir BenMohamed

**Affiliations:** 1 Laboratory of Cellular and Molecular Immunology, Gavin Herbert Eye Institute, School of Medicine, University of California Irvine, Irvine, CA 92697, USA; aalamich@uci.edu (A.A.C.); akhan@uci.edu (A.A.K.); rsrivas@uci.edu (R.S.); skaran1@hs.uci.edu (S.K.); ylekbach@hs.uci.edu (Y.L.); hvahed@uci.edu (H.V.); 2 Department of Molecular Biology & Biochemistry, University of California Irvine, Irvine, CA 92697, USA; 3 Institute for Immunology, School of Medicine, University of California Irvine, Irvine, CA 92697, USA

**Keywords:** ocular herpes, cornea, trigeminal ganglia, CD8^+^ T cells, CD57, senescent

## Abstract

Herpes simplex virus (HSV)-specific CD8^+^ T cells protect mice from herpes infection and disease. However, the phenotype and function of HSV-specific CD8^+^ T cells that play a key role in the “natural” protection seen in HSV-1-seropositive healthy asymptomatic (ASYMP) patients (who have never had clinical herpes disease) remain to be determined. We previously reported that symptomatic (SYMP) patients (who have frequent bouts of recurrent ocular herpes disease) had more undifferentiated and dysfunctional HSV-specific CD8^+^ T cells. In contrast, healthy ASYMP individuals maintained a significantly higher proportion of differentiated polyfunctional CD8^+^ T cells. Here, we report that HSV-specific CD8^+^ T cells from 10 SYMP patients, but not HSV-specific CD8^+^ T cells from 10 ASYMP patients, have phenotypic and functional characteristics of cellular senescence, including: (i) high frequency of senescent (CD57^+^) and exhausted (PD-1^+^) CD8^+^ T cells; (ii) late terminally differentiated (KLRG1^+^), non-proliferating CD8^+^ T cells; (iii) HSV-specific CD8^+^ T cells which decreased in number over time and were not homeostatically maintained, as indicated by a reduction in the number of CD127^+^CD8^+^ T cells; (iv) loss of the co-stimulatory molecule CD28 on HSV-specific CD8^+^ T cells; and (v) decreased production of effector molecules (granzyme B and perforin) by HSV-specific CD8^+^ T cells. Our findings provide insights into the role of senescence in HSV-specific CD8^+^ T cells in susceptibility to recurrent herpes and have implications for T-cell-based immunotherapeutic strategies against recurrent herpes in humans.

## 1. Introduction

Herpes Simplex Virus type 1 (HSV-1) infects over a billion individuals worldwide, causing a wide range of mild to life-threatening diseases [1,2]. Although the virus reactivates from latency and is shed multiple times each year in body fluids (i.e., tears, saliva, nasal and vaginal secretions), most reactivations are subclinical due to efficient immune-mediated control of infection and disease [3,4,5,6]. Thus, the majority of infected individuals remain asymptomatic (ASYMP) and do not develop recurrent herpetic diseases (e.g., cold sores, genital or ocular herpetic disease). However, a small proportion of individuals experience endless recurrences of herpetic disease, usually multiple times a year, often requiring continuous antiviral therapy (i.e., acyclovir and derivatives) [7,8]. In symptomatic (SYMP) individuals, HSV-1 frequently reactivates from latency, re-infects the eyes, and may trigger recurrent and severe corneal herpetic disease, a leading cause of infectious corneal blindness in the industrialized world [9,10,11]. In the United States, up to 450,000 individuals have a history of recurrent herpetic stromal keratitis (rHSK), a T cell-mediated immunopathological lesion of the cornea [9,10,11]. Thus, gaining a better understanding of the immune mechanisms that confer protection from HSV-1 infection and disease is highly desirable for developing more effective vaccines and immunotherapies to reduce HSV-1-related diseases.

The primary factors that influence the reactivation of HSV virus in ASYMP participants without developing any disease throughout their lifespan remain an active field of investigation. The present study sought to determine whether differences in the maintenance of optimal CD8^+^ T cell responses exist between HSV-1 seropositive ASYMP and SYMP participants. The immunosenescence exhibited by a progressive increase in the number of memory CD8^+^ T cells shows poor functionality in terms of killing persistent virus [12]. We hypothesize that the repeated stimulation of the CD8^+^ T cells by frequent HSV1 reactivation in SYMP individuals leads to immunosenescence, but ASYMP individuals maintain functional CD8^+^ T cell responses. We investigated the association of key CD8^+^ T cells with markers of immune differentiation (CD28), senescence (KLRG-1 and CD57), and survival of HSV-specific CD8^+^ T cells. In the literature, CD57^+^CD8^+^ T cells have been associated with various chronic inflammatory diseases including HIV and other persistent infections [13,14,15,16,17]. However, their pathogenic roles have not yet been elucidated in HSV-1 seropositive SYMP participants. This study represents a step forward in assessing the immunological characteristics of CD57^+^CD8^+^ T cells in ASYMP and SYMP participants.

The differentiation of T cells toward a senescent phenotype is typically characterized by clonal expansion of CD8^+^ T cells lacking CD28 expression [13,18]. Recent studies have shown that CD8^+^CD28^−^CD57^+^ T cells occupy critical immunological space, rendering them inaccessible to the naive and early memory T-cells. This phenomenon has been observed in the early stage of HIV infection with more rapid progression to AIDS [13,14,16,19,20]. Expression of KLRG1 identifies the subset of CD8^+^ T cells in humans that are capable of secreting cytokines but fail to proliferate after stimulation, thereby preventing further clonal expansion [21,22]. An increased frequency of late-senescent CD8^+^ T cells with reduced surface expression of CD28 results in defective proliferation abilities. The absence of survival marker CD127 (IL-7R) results in defective killing abilities, and reduced surface expression of CD28 leads to defective proliferation abilities. Here, we demonstrate that CD8^+^ T cells from SYMP individuals express a higher percentage of CD57 and KLRG1 and a lower percentage of CD28 and CD127. The loss of CD127 in HSV-specific CD8^+^ T cells could lead to apoptosis, which may impair homeostatic maintenance. The T-box transcription factor T-bet plays a crucial role in determining the differential fate of CD8^+^ T cells responding to infection, optimal memory, and terminal differentiation. In CD8^+^ T cells, T-bet is upregulated upon activation and is associated with the induction of effector functions, including cytotoxicity. The expression of T-bet was increased in CD8^+^ T cells from SYMP individuals and correlated closely with the expression of CD57 and KLRG1. Although senescent CD8^+^ T cells typically preserve effector function (i.e., the ability to produce cytokines and even kill target cells), their limited proliferative potential impairs their ability to mount robust immune responses and expand upon reactivation. During HIV infection, senescent CD57^+^CD8^+^ T cells produce IFN but exhibit poor proliferative ability and increased sensitivity to activation-induced cell death. Thus, the accumulation of senescent CD8^+^ T cells contributes to the deterioration of protective immune responses.

In this report, we found that HSV-1 seropositive SYMP individuals exhibit an increased frequency of senescent CD8^+^ T cells which lack co-stimulatory molecule CD28 and survival molecule CD127 as compared to ASYMP participants. We also show that higher expression of transcription factor T-bet is associated with increased co-expression of CD57 and KLRG1. Thus, cellular senescence appears to be a major feature of HSV-specific CD8^+^ T cells in the SYMP individuals, and T-bet is likely involved in the underlying molecular regulation of this terminally differentiated state. Altogether, these results indicate a significantly higher frequency of HSV-specific senescent CD8^+^ T cells in SYMP individuals which lack proliferative capacity and co-stimulatory markers and cannot maintain homeostatic proliferation, in contrast to those in HSV-seropositive ASYMP individuals. These findings have important implications for developing T-cell-based immunotherapeutic strategies for recurrent herpes in humans.

## 2. Materials and Methods

### 2.1. Human Study Population

Over the last roughly 13 years (i.e., January 2003 to May 2016), we have screened 781 individuals for HSV-1 and HSV-2 seropositivity (Table 1). Five hundred forty-three individuals were White, 238 were non-White (African, Asian, Hispanic, and others), 395 were females, and 386 were males. Among these, a cohort of 283 immuno-competent individuals, with an age range of 21–67 (median 30), who were seropositive for HSV-1 and seronegative for HSV-2, were enrolled in the present study. All patients were negative for HIV and HBV and had no history of immunodeficiency. In total, 680 patients were HSV-1, HSV-2, or HSV-1/HSV-2 seropositive, among which 711 patients were healthy and ASYMP (individuals who, in the absence of therapy, had never had any recurrent herpes disease, ocular, genital, or elsewhere, based on their self-report and physician examination. Even a single episode of any herpetic disease in their lifespan would exclude the individual from this group). The remaining 70 patients were defined as HSV-seropositive SYMP who suffered from frequent and severe recurrent genital, ocular, and/or oro-facial lesions, with two patients having had clinically well-documented repetitive herpes stromal keratitis (HSK), including one patient with ~20 episodes over 20 years, which necessitated several corneal transplantations.

Signs of recurrent disease in SYMP patients were defined as herpetic lid lesions, herpetic conjunctivitis, dendritic or geographic keratitis, stromal keratitis, and iritis consistent with HSK, with one or more episodes per year for the past 2 years. However, at the time of blood collection, SYMP patients had no recurrent disease (other than corneal scarring) and had no recurrences in the past 30 days. They had no ocular disease other than HSK, no history of recurrent genital herpes, and were HSV-1 seropositive and HSV-2 seronegative. Because the spectrum of recurrent ocular herpetic disease was wide, our emphasis was mainly on the number of recurrent episodes and not on the severity of the recurrent disease. No attempt was made to assign specific T cell epitopes to the specific severity of recurrent lesions. Patients were also excluded if they: (1) had an active ocular (or elsewhere) herpetic lesion or had had one in the past 30 days; (2) were seropositive for HSV-2; (3) were pregnant or breastfeeding; or (4) were on acyclovir or other related anti-viral drugs or any immunosuppressive drugs at the time of blood draw. SYMP and ASYMP groups (*n* = 10 participants per group) were matched for age, gender, serological status, and race. Eighty-six healthy control individuals were seronegative for both HSV-1 and HSV-2 and had no history of ocular herpes, genital lesions, or oro-facial herpes disease. All participants were enrolled at the University of California Irvine under approved Institutional Review Board-approved protocols (IRB#2003-3111 and IRB#2009-6963). Written informed consent was received from all participants before inclusion in the study.

### 2.2. HLA Typing

HLA-A2 sub-typing was performed using a commercial Sequence-Specific Primer (SSP) kit, following the manufacturer’s instructions (SSPR1-A2; One Lambda, Canoga Park, Los Angeles, CA, USA). Briefly, genomic DNA extracted from PBMC of HSV-seropositive SYMP and ASYMP individuals was analyzed using a TECAN DNA workstation from a 96-well plate with 2 L volume per well, as previously described [23]. The yield and purity of each DNA sample were tested using a UV-spectrophotometer. The integrity of DNA samples was ascertained by electrophoresis on an agarose gel. Each DNA sample was then subjected to multiple small-volume PCR reactions using primers specific to areas of the genome surrounding the single-point mutations associated with each allele. Only primers that matched the specific sequence of a particular allele would amplify a product. The PCR products were subsequently electrophoresed on a 2.5% agarose gel with ethidium bromide, and the pattern of amplicon generation was analyzed using HLA Fusion Software (One Lambda, Inc., Canoga Park, Los Angeles, CA, USA). In addition, the HLA-A2 status was confirmed by staining PBMC with anti-HLA-A2 mAb, BB7.2 (BD Pharmingen, San Diego, CA, USA), at 4 °C for 30 min. The cells were washed, placed on a BD LSR II, and analyzed using FlowJo software version 9.7.5 (TreeStar).

### 2.3. Peripheral Blood Mononuclear Cell (PBMC) Isolation

Individuals (negative for HIV, HBV, and with or without any HSV infection history) were recruited at Gavin Herbert Eye Institute and UCI Institute for Clinical and Translational Science (ICTS). Between 40 and 100 mL of blood were drawn into yellow-top Vacutainer^®^ Tubes (Becton Dickinson, Franklin Lakes, NJ, USA). The serum was isolated and stored at −80 °C for the detection of anti-HSV-1 and HSV-2 antibodies, as described earlier [8]. PBMC were isolated by gradient centrifugation using a leukocyte separation medium (Cellgro, Innovation, VA, USA). The cells were washed in PBS and re-suspended in a complete culture medium consisting of RPMI-1640, 10% FBS (Bio-Products, Woodland, Vacaville, CA, USA) supplemented with 1× penicillin/L-glutamine/streptomycin, 1× sodium pyruvate, 1× non-essential amino acids, and 50 μM of 2-mercaptoethanol (Life Technologies, Rockville, MD, USA). Freshly isolated PBMCs were also cryo-preserved in 90% FCS and 10% DMSO in liquid nitrogen for future testing.

### 2.4. Flow Cytometry Analysis

PBMCs were analyzed by flow cytometry after staining with fluorochrome-conjugated human and mouse-specific monoclonal antibodies (mAbs). The following anti-human antibodies were used: CD3 (clone SK7) PE-Cy7, CD44 (clone G44-26) A700, CD8 (clone SK1) APC-Cy7, T-bet (clone O4-46) Alexa Fluor 488 (BD Pharmingen); Ki-67 (clone 20Raj1) PE-Cy7, Eomes (clone WD1928) eFluor 660 (eBioscience). CD28 (clone CD28.2) A700; and CD57 (clone HCD57) FITC; KLRGI (clone 2F1/KLRG1) APC (BioLegend). The glycoprotein gB tetramers were made by NIH Tetramer Core Facility, Emory University, Atlanta, GA. For surface staining, mAbs against various cell markers were added to a total of 1 × 10^6^ PBMC in phosphate-buffered saline containing 1% FBS and 0.1% sodium azide (FACS buffer) for 45 min at 4 °C. After washing with FACS buffer, cells were permeabilized for 20 min on ice using the Cytofix/Cytoperm Kit (BD Biosciences) and then washed twice with Perm/Wash Buffer (BD Bioscience). Intra-cellular cytokine/intra-nuclear transcription factor-staining mAbs were then added to the cells and incubated for 45 min on ice in the dark. Cells were washed again with Perm/Wash and FACS Buffer and fixed in PBS containing 2% paraformaldehyde (Sigma-Aldrich, St. Louis, MO, USA). For each sample, 200,000 total events were acquired on BD LSRII. Antibody capture beads (BD Biosciences) were used as individual compensation tubes for each fluorophore in the experiment. To define positive and negative populations, we employed fluorescence minus controls for each fluorophore used in this study, when initially developing staining protocols. In addition, we further optimized gating by examining known negative cell populations for background-level expression. The gating strategy was similar to that used in our previous work [5]. Briefly, we gated on lymphocytes, single cells, CD3^+^ cells, and CD8^+^ cells before finally gating human epitope-specific CD8^+^ T cells using gB epitope-specific tetramers (Supplementary Figure S1). Data analysis was performed using FlowJo version 9.7.5 (TreeStar, Ashland, OR, USA). Statistical analyses were done using GraphPad Prism version 5 (La Jolla, San Diego, CA, USA).

### 2.5. CD107 Cytotoxicity Assay

To detect gB-specific cytolytic CD8^+^ T cells in PBMC, an intracellular CD107^a/b^ cytotoxicity assay was performed as described by Betts et. al. [24] with a few modifications. Briefly, 1 × 10^6^ PBMCs from patients were transferred into 96-well flat bottom plates and stimulated with immunodominant and subdominant gB peptide epitopes (10 μg/mL in 200 μL complete culture medium) in the presence of anti-CD107^a^-FITC, CD107^b^-FITC, and BD Golgi stop (10 μg/mL) for 6 h at 37 °C. PHA (10 μg/mL) (Sigma); no peptide were used as positive and negative controls, respectively. At the end of the incubation period, the cells were transferred to 96-well round bottom plate and washed with FACS buffer. Anti-CD107^a^-FITC and anti-CD107^b^-FITC were used for surface stain and IFN was detected using intra-cellular staining as described above.

### 2.6. Statistical Analyses

Data for each assay were compared by analysis of variance (ANOVA) and Student’s *t*-test using Graph Pad Prism 5 software version 9.5.0 (San Diego, CA, USA). Differences between the groups were identified by ANOVA, and multiple comparison procedures, as we previously described in [25]. Data are expressed as the mean ± SD. Results were considered statistically significant at *p* < 0.05.

## 3. Results

### 3.1. Decline of HSV-Specific CD8^+^ T Cells in Seropositive SYMP Individuals

The characteristics of the SYMP and ASYMP study population used in the present study, concerning gender, age, HLA-A*02:01 frequency distribution, HSV-1/HSV-2 seropositivity, and the status of ocular herpetic disease are presented in Table 1 and are detailed in the Materials and Methods. Since HSV-1 is the main cause of ocular herpes, only individuals who were HSV-1 seropositive and HSV-2 seronegative were enrolled in this study. The HSV-1 seropositive participants were segregated into two groups: (*n* = 10 participants per group): (i) HLA-A*02:01 positive, HSV-1-infected ASYMP participants who had never experienced any clinically detectable herpes disease; and (ii) HLA-A*02:01 positive HSV-1-infected SYMP participants with a history of numerous episodes of well-documented recurrent clinical herpes diseases, such as herpetic lid lesions, herpetic conjunctivitis, dendritic or geographic keratitis, stromal keratitis, and iritis consistent with rHSK, with one or more episodes per year for the past 2 years. Only SYMP patients who were not on acyclovir or other anti-viral or anti-inflammatory drug treatments at the time of blood sample collections were enrolled.

**Figure 1:**
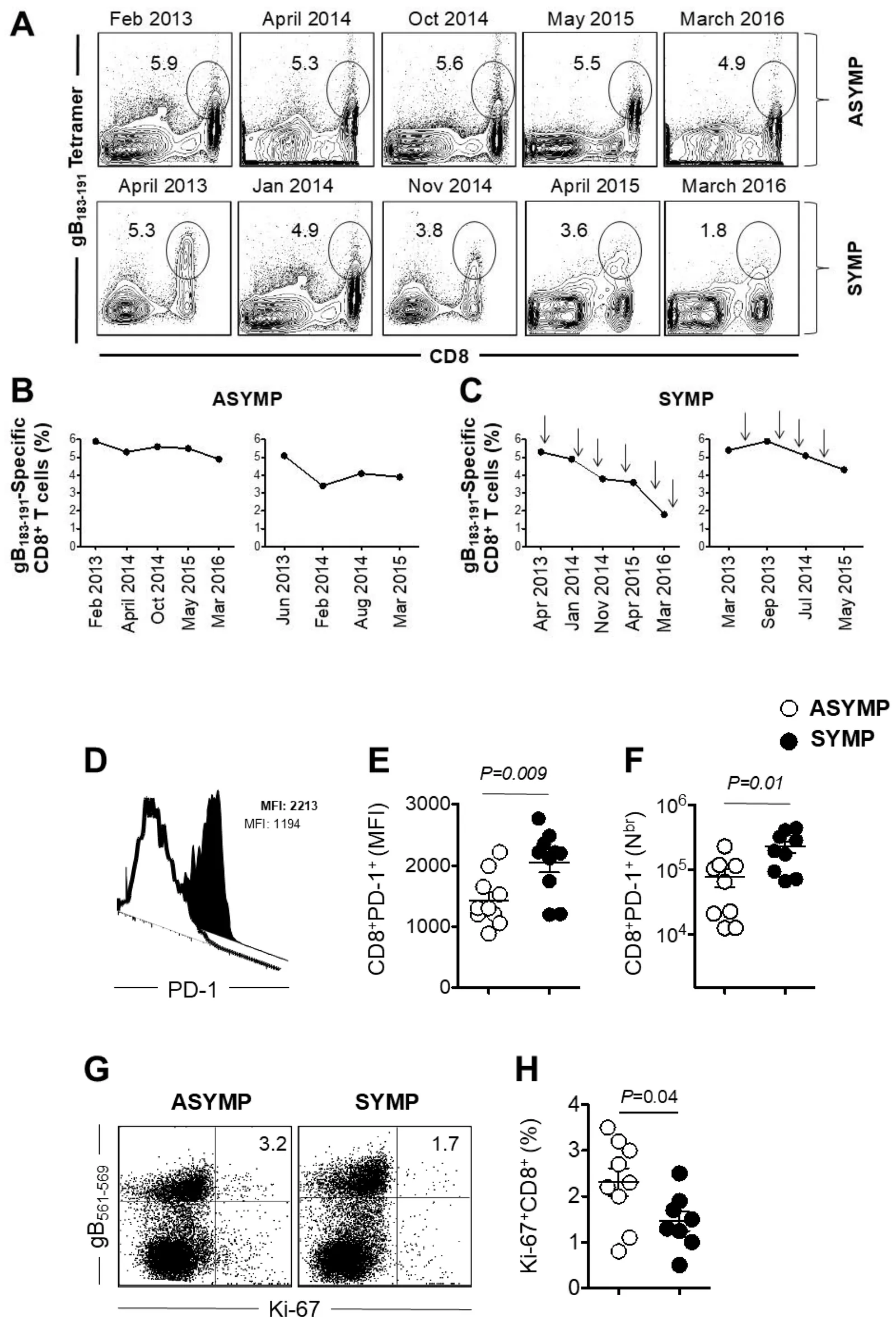
The decline of gB_183–191_-specific CD8^+^ T cells in SYMP individuals but not in ASYMP individuals. gB_183–191_-specific CD8^+^ T cells were measured in HLA-A*02:01-positive, HSV-1 seropositive SYMP and ASYMP individuals at different time points. Each time history of recurrent disease was recorded. (**A**) The frequency of gB_183–191_-specific CD8^+^ T cells is shown as FACS dot plots. The top panel shows a dot plot of gB_183–191_-specific CD8^+^ T cells at the different time points of ASYMP individuals. The bottom panel shows a dot plot of gB_183–191_-specific CD8^+^ T cells at the different time points of SYMP individuals. (**B**) The line graph shows the kinetic of gB_183–191_-specific CD8^+^ T cells at different time points of ASYMP individuals. The frequency of the gB_183–191_-specific CD8^+^ T cells was maintained over time. (**C**) The line graph shows the kinetic of gB_183–191_-specific CD8^+^ T cells at the different time points of SYMP individuals. Unlike ASYMP individuals, the frequency of the gB_183–191_-specific CD8^+^ T cells declined over time. The arrow on the line graph shows the frequency of recurrent disease in SYMP individuals. (**D**) The representative histogram of PD-1 expression on gB_183–191_-specific CD8^+^ T cells. A filled histogram represents an expression of PD-1 in SYMP individuals, and an open histogram represents ASYMP individuals. (**E**) Expression level and (**F**) The absolute number of PD-1 was significantly higher in SYMP individuals compared to ASYMP individuals. (**G**) Representative dot plot of gB_183–191_-specific CD8^+^ T cells stained for proliferation marker Ki-67. (**H**) Frequency of Ki-67^+^CD8^+^ T cells gated on gB_183–191_ tetramer. Open circles indicate data for ASYMP individuals and filled circles represent data for SYMP individuals. The results are representative of two independent experiments for each individual. The indicated *p* values, calculated using one-way ANOVA, show the statistical significance of differences between SYMP and ASYMP individuals. The data are representative of two independent experiments, and the bars represent SD between the experiments.

We monitored in the peripheral blood the frequency of HSV gB_183–191_ epitope specific CD8^+^ T cells using gB_183–191_ loaded HLA-A*02:01 specific tetramers for more than 3 years in HLA-A*02:01 positive, HSV-1 seropositive ASYMP (*n* = 10), and SYMP participants (Figure 1). At each time point, history of recurrent disease was recorded. Arrow shows the frequency of recurrent disease. The frequency of gB_183–191_-specific CD8^+^ T cells are shown as FACS dot plots (Figure 1A). The top panel shows dot plot of gB_183–191_-specific CD8^+^ T cells at different time points of representative ASYMP participants. The bottom panel shows dot plots of gB_183–191_-specific CD8^+^ T cells at different time points of representative SYMP participants. The line graph (Figure 1B) shows the kinetic of gB_183–191_-specific CD8^+^ T cells at different time points of two representative ASYMP individuals. In both ASYMP participants, the frequency of the CD8^+^ T cells was maintained over time. In one ASYMP participant, the frequency of the gB_183–191_-specific CD8^+^ T cells was very similar (4.9% to 5.9%) during this time period. However, initially, in one ASYMP participant, there was a drop in the frequency of the gB_183–191_-specific CD8^+^ T cells (from 5.1% to 3.2%), which was subsequently maintained for another two years (3.2% to 3.8%). The line graph (Figure 1C) shows the change over time of gB_183–191_-specific CD8^+^ T cells of two representative SYMP participants. Unlike ASYMP participants, the frequency of the gB_183–191_-specific CD8^+^ T cells declined over time. In one SYMP participant, there was a sharp decline in gB_183–191_-specific CD8^+^ T cells (5.3% to 1.8%). However, in another SYMP individual, there was a moderate decline in the gB_183–191_-specific CD8^+^ T cells (from 5.4% to 4.2%). Each arrow on the line graph shows an episode of the recurrent disease in SYMP participants. We next determined the frequency of HSV-specific senescent CD8^+^ T cells by measuring the expression of CD57 PMBC isolated from HSV-1 seropositive ASYMP and SYMP participants. We also found that the gB_183–191_-specific CD8^+^ T cells from SYMP individuals were exhausted. Figure 1D shows a representative histogram of PD-1 expression on gB_183–191_-specific CD8^+^ T cells (a filled histogram represents an expression of PD-1 in SYMP participants, and an open histogram represents ASYMP individual). Expression level (Figure 1E) and the absolute number of (Figure 1F) of PD-1 were significantly higher in SYMP individuals compared to ASYMP participants.

Further, we evaluated the proliferative capacity of CD8^+^ T cells from SYMP and ASYMP participants using intra-nuclear staining of Ki-67 to see if there was any difference in the recent proliferation of CD8^+^ T cells. Figure 1G is a representative dot plot of CD8^+^ T cells stained for proliferation marker Ki-67. The frequency of Ki-67^+^CD8^+^ T cells gated on gB_183–191_ tetramer showed significantly (*p* = 0.04) less proliferation of CD8^+^ T cell isolated from SYMP vs. ASYMP participants. In SYMP participants, a median frequency of Ki-67^+^CD8^+^ T was recorded as 2.3%, whereas in ASYMP participants, it was recorded as 1.3% (Figure 1H). Open circles indicate data for ASYMP participants and filled circles represent data for SYMP participants. The results are representative of two independent experiments for each participant. Altogether, these data indicated a higher frequency of terminally differentiated gB_183–191_-specific CD8^+^ T cells which had lost proliferative capacity in SYMP than in ASYMP participants. Unlike with ASYMP participants, HSV-specific CD8^+^ T cells from SYMP participants were not maintained and remained functionally exhausted. Next, we aimed to evaluate whether the senescence could cause a decline in the frequency of CD8^+^ T cells in SYMP individuals but not in ASYM participants.

### 3.2. Increased Frequency of Senescent CD8^+^ T Cells in HSV-Seropositive SYMP Participants Compared to ASYMP Participants

**Figure 2:**
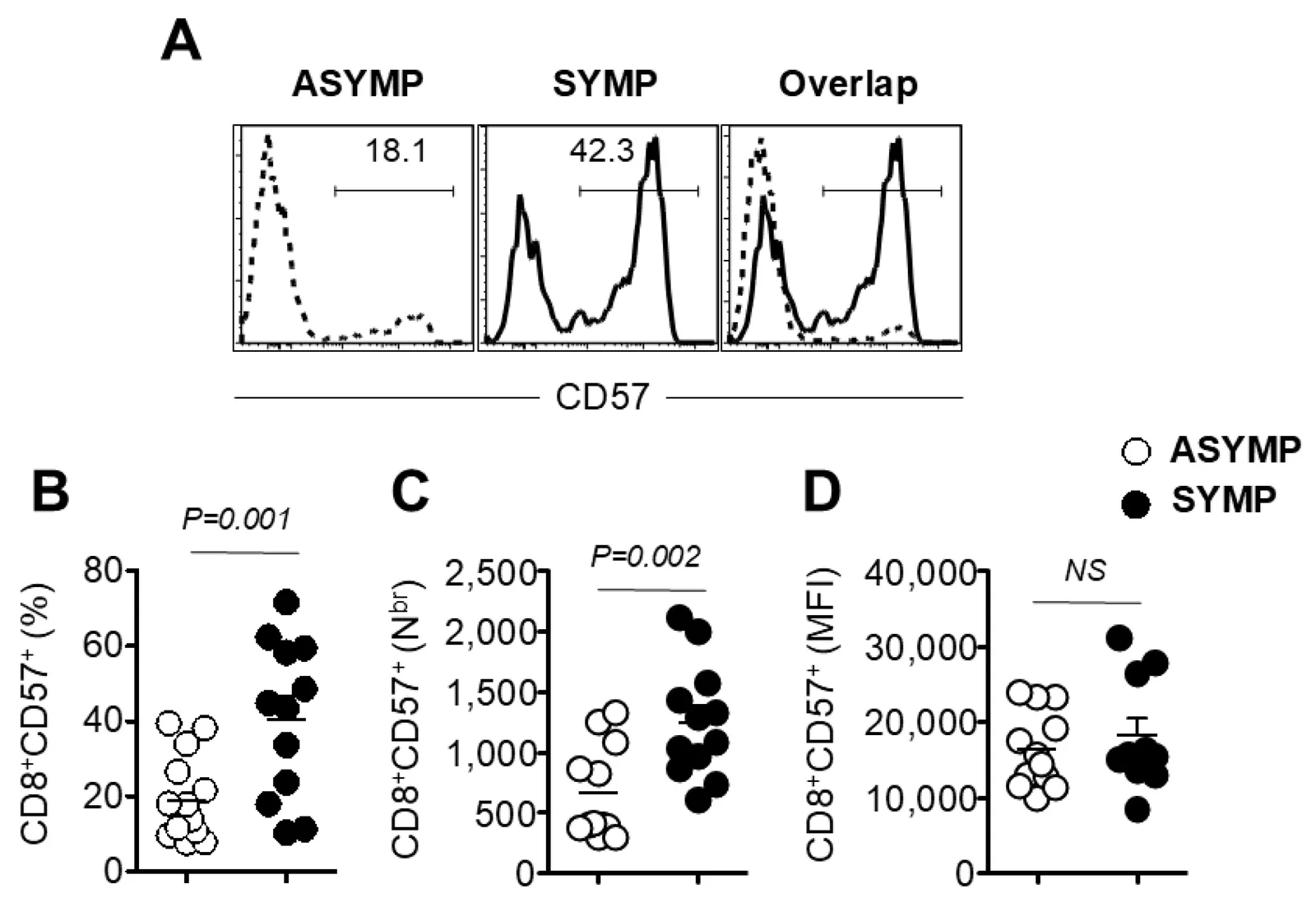
Increased frequency of senescent CD8^+^ T cells in SYMP individuals compared to ASYMP individuals. PBMC isolated from HLA-A*02:01-positive, HSV-1 seropositive SYMP and ASYMP individuals was stained for senescent marker CD57. (**A**) Representative histogram of CD57 expression on gB_183–191_-specific CD8^+^ T cells. (**B**) Frequency of CD8^+^CD57^+^ on gB_183–191_-specific CD8^+^ T cells. (**C**) The absolute number of CD8^+^CD57^+^ on gB_183–191_-specific CD8^+^ T cells. (**D**) Mean Fluorescent Intensity (MFI) of CD57 expression gated on gB_183–191_-specific CD8^+^ T cells. Open circles indicate data for ASYMP individuals and filled circles represent data for SYMP individuals.

Next, we investigated the role of senescent CD8^+^ T cells in SYMP vs. ASYMP subjects. PBMC isolated from HLA-A*02:01-positive, HSV-1 seropositive SYMP and ASYMP participants was stained for senescent marker CD57 on HSV-specific CD8^+^ T cells. Figure 2A shows a representative histogram of CD57 expression on gB_183–191_-specific CD8^+^ T cells from ASYMP participants. The right panel shows the overlap of CD57 expression between ASYMP and SYMP participants. The frequency of CD8^+^CD57^+^ on gB_183–191_-specific CD8^+^ T cells from ASYMP and SYMP individuals is shown in Figure 2B. There was a significantly (*p* = 0.001) higher percentage of gB_183–191_-specific CD8^+^ T cells expressing CD57 in SYMP individuals than in ASYMP participants. The median expression of CD57 on gB_183–191_-specific CD8^+^ T cells isolated from SYMP individuals was 42.3%, whereas it was 18.1% in ASYMP individuals. The absolute number of CD8^+^CD57^+^ on gB_183–191_-specific CD8^+^ T cells is shown in Figure 2C. We observed a significantly (*p* = 0.002) higher number of gB_183–191_-specific CD8^+^ T isolated from SYMP than ASYMP individuals. As shown in Figure 2D, no significant difference was observed in the Mean Fluorescent Intensity (MFI) of CD57 expression gated on gB_183–191_-specific CD8^+^ T cells from SYMP and ASYMP individuals. However, an increased level of CD57 expression was observed in SYMP participants compared to ASYMP participants. Open circles indicate data for ASYMP individuals and filled circles represent data for SYMP participants. The results are representative of two independent experiments for each participant.

**Figure 3:**
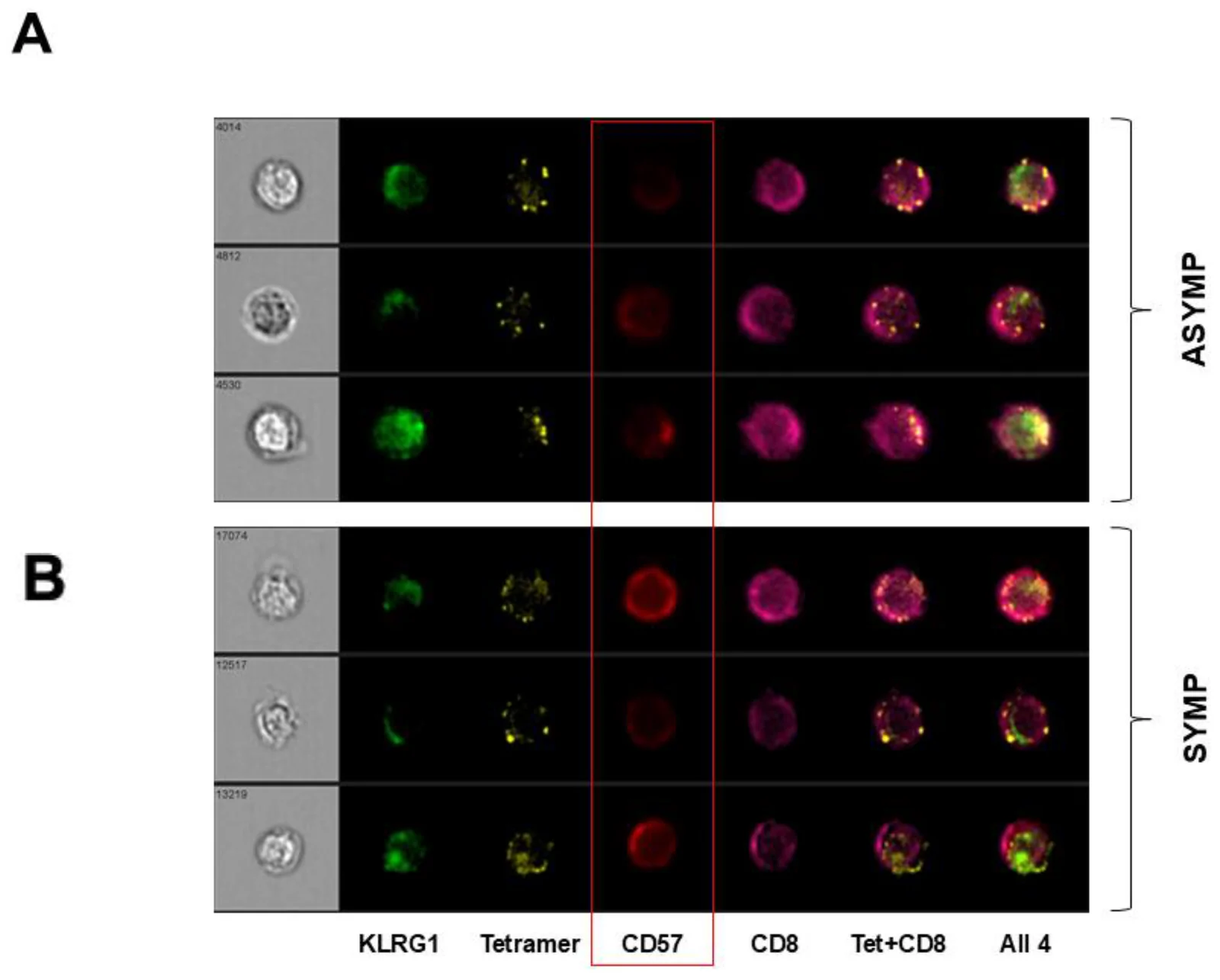
Increased expression of CD57 on gB_183–191_-specific CD8^+^ T cells in SYMP individuals compared to SYMP individuals. PBMC isolated from HLA-A*02:01-positive, HSV-1 seropositive SYMP and ASYMP individuals was stained for senescent marker CD57 along with CD8, gB_183–191_ tetramer, and KLRG-1. Images of individual PBMC stained with different markers were visualized and captured using Image Stream. Data were analyzed using the software IDEA version 4. Representative images of individual PMBC isolated from HSV-1 seropositive (**A**) ASYMP patients and (**B**) SYMP patients. Cells are shown as individually stained with KLRG-1, gB_183–191_ tetramer, CD57, CD8, gB_183–191_ tetramer, and CD8, and superimposed image of PBMC stained with KLRG-1, gB_183–191_ tetramer, CD57, and CD8^+^ T cells.

Next, we observed the expression of CD57 on gB_183–191_-specific CD8^+^ T from SYMP and ASYMP participants using a powerful tool for capturing images stained with fluorescent mAbs (ImageStream). PBMC was stained with different surface markers (KLRG-1, gB_183–191_ tetramer, CD57, and CD8), similar to FACS staining, as outlined in the Materials and Methods section. An image of each cell was captured using ImageStream (EMD Millipore) and data were analyzed using the IDEA V4 software. Figure 3 shows an image of an individual cell showing expression of KLRG-1, gB_183–191_ tetramer, CD57, CD8^+^, and superimposed image of all four mAbs on PBMC isolated from HSV-1 seropositive ASYMP (Figure 3A) and SYMP individuals (Figure 3B). As shown in ImageStream, the expression of CD57 was higher in PBMC isolated from SYMP than from ASYMP participants. However, no differences were observed in the staining of gB_183–191_ tetramer and CD8^+^ T cells in PBMC isolated from both groups. Altogether, we detected a significantly higher expression of CD57 in SYMP participants compared to ASYMP participants.

### 3.3. HSV-Specific CD8^+^ T Cells Isolated from SYMP Participants Were Terminally Differentiated

**Figure 4:**
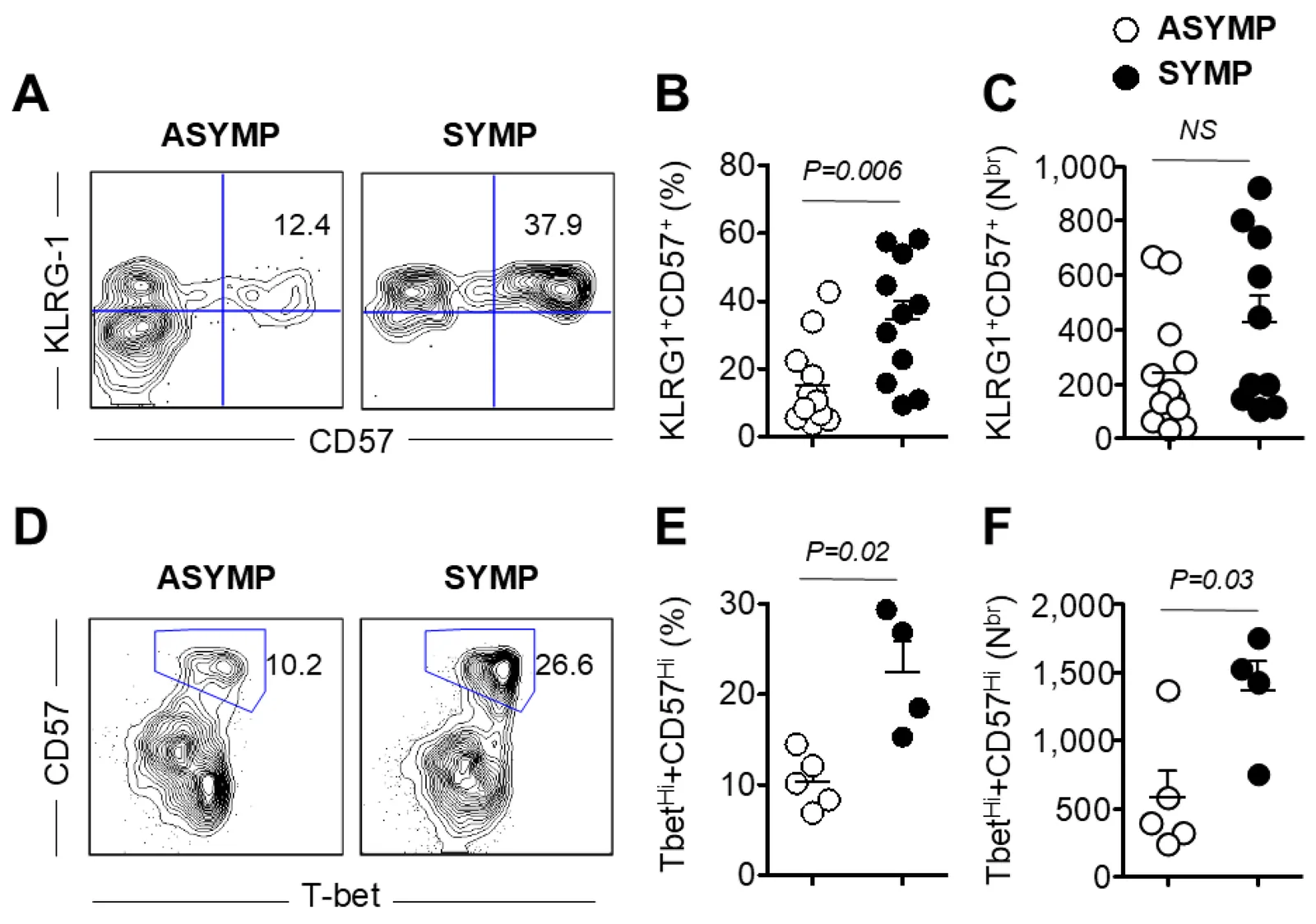
HSV-1-specific CD8^+^ T cells from SYMP individuals were terminally differentiated and could not divide. PBMC isolated from HLA-A*02:01-positive, HSV-1 seropositive SYMP and ASYMP individuals was stained for the terminally differentiated marker KLRG-1 and transcription factor T-bet, along with CD57. Cells were also stained for Ki-67 to see the percentage of proliferating cells. (**A**) Representative contour plot of CD57 expression on terminally differentiated CD8^+^ T cells gated on gB_183–191_ tetramer in PBMC isolated from SYMP and ASYMP individuals. (**B**) Frequency of KLRG-1^+^CD57^+^ on gB_183–191_-specific CD8^+^ T cells. (**C**) The absolute number of KLRG-1^+^CD57^+^ on gB_183–191_-specific CD8^+^ T cells. (**D**) A representative contour plot of T-betHi^+^CD57^+^ expression gated on gB_183–191_ tetramer in PBMC isolated from SYMP and ASYMP individuals. (**E**) Frequency of T-betHi^+^CD57^+^ on gB_183–191_-specific CD8^+^ T cells. (**F**) The absolute number of T-betHi^+^CD57^+^ on gB_183–191_-specific CD8^+^ T cells. Open circles indicate data for ASYMP individuals and filled circles represent data for SYMP individuals. The results are representative of two independent experiments for each individual. The indicated *p* values, calculated using one-way ANOVA, show the statistical significance of differences between SYMP and ASYMP individuals. The data are representative of two independent experiments, and the bars represent SD between the experiments.

Subsequently, we studied the expression of markers expressed on terminally differentiated gB_183–191_ tetramer-specific CD8^+^ T cells. Since terminally differentiated CD8^+^ T cells do not proliferate, we stained cells with Ki-67 to study proliferative capacity. A representative contour plot of KLRG-1^+^CD57^+^ expression on terminally differentiated gB_183–191_-specific CD8^+^ T cells isolated from SYMP and ASYMP individuals is shown in Figure 4A. The frequency of KLRG-1^+^CD57^+^ on gB_183–191_-specific CD8^+^ T cells isolated from SYMP individual (37.9%) was significantly (*p* = 0.006) higher compared to ASYMP (12.4%), as shown in Figure 4B. The absolute number of KLRG-1^+^CD57^+^ expressing gB_183–191_-specific CD8^+^ T cells was higher for SYMP compared to ASYMP (Figure 4C). Though the difference in absolute number was not significant in SYMP vs. ASYMP individuals, PBMC isolated from HLA-A*02:01-positive, HSV-1 seropositive SYMP and ASYMP individuals was stained for transcription factor T-bet along with the marker of senescent CD57 on gB_183–191_-specific CD8^+^ T cells isolated from SYMP and ASYMP individuals. Figure 4D shows a contour plot of T-betHi^+^CD57Hi^+^ gated on gB_183–191_-specific CD8^+^ T cells in PBMC isolated from SYMP and ASYMP individuals. We observed a significant difference (*p* = 0.02) in the frequency of T-bet^Hi^CD57^Hi^ gated on gB_183–191_-specific CD8^+^ T cells isolated from SYMP as compared to ASYMP individuals. The median frequency of T-bet^Hi^CD57^Hi^ gated on gB_183–191_-specific CD8^+^ T cells in SYMP individuals was 26.6%, whereas in ASYMP individuals, it was recorded as 10.2% (Figure 4E). We also observed a significant (*p* = 0.03) difference in the absolute number of T-bet^Hi^CD57^Hi^ gated on gB_183–191_-specific CD8^+^ T cells. SYMP individuals harbored a higher absolute number of T-bet^Hi^CD57^Hi^ gated on gB_183–191_-specific CD8^+^ T cells compared to ASYMP individuals (Figure 4F). Open circles indicate data for ASYMP individuals, while filled circle represent data for SYMP individuals. The results are representative of two independent experiments for each individual.

### 3.4. Decreased Expression of Co-Stimulatory Molecule CD28 and Survival Molecule CD127 on PBMC Isolated from HSV-Seropositive SYMP Participants

**Figure 5:**
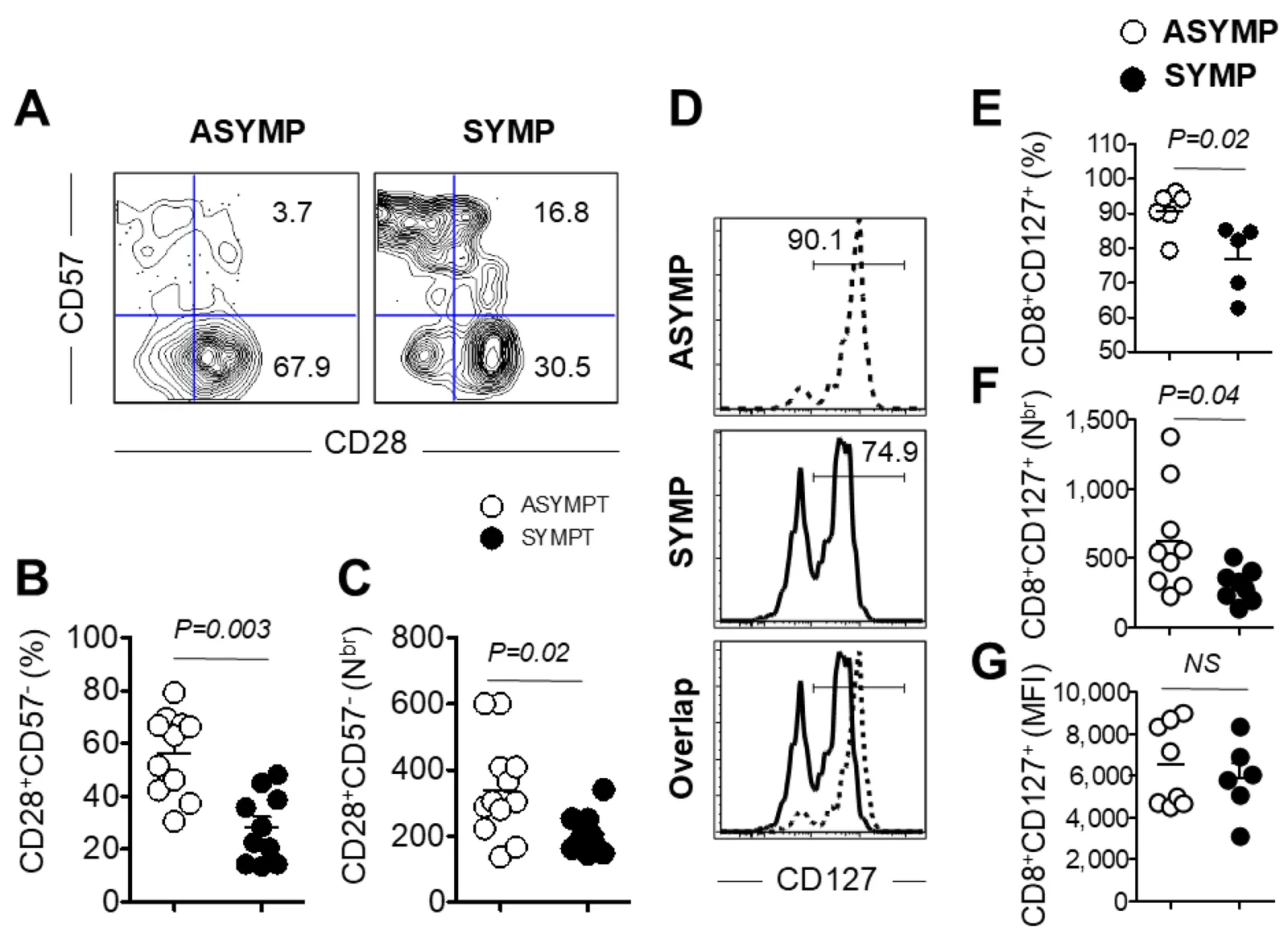
Decreased expression of co-stimulatory molecule CD28 and survival molecule CD127 on PBMC isolated from SYMP individuals. PBMC isolated from HLA-A*02:01-positive, HSV-1 seropositive SYMP and ASYMP individuals was stained for co-stimulatory and survival molecules. (**A**) Representative contour plot of CD28 and CD57 expression gated on gB_183–191_ tetramer in PBMC isolated from SYMP and ASYMP individuals. (**B**) Frequency of CD28^+^CD57^−^ on gB_183–191_-specific CD8^+^ T cells. (**C**) The absolute number of CD28^+^CD57^−^ on gB_183–191_-specific CD8^+^ T cells. (**D**) Representative histogram showing the expression level of survival molecule CD127 gated on gB_183–191_-specific CD8^+^ T cells. (**E**). Frequency of CD127 gated on gB_183–191_-specific CD8^+^ T cells. (**F**) The absolute number of CD127 gated on gB_183–191_-specific CD8^+^ T cells. (**G**) Mean Fluorescent Intensity (MFI) of CD127 expression on gB_183–191_-specific CD8^+^ T cells. Open circles indicate data for ASYMP individuals and filled circles represent data for SYMP individuals. The results are representative of two independent experiments for each individual. The indicated *p* values, calculated using one-way ANOVA, show the statistical significance of differences between SYMP and ASYMP individuals. The data are representative of two independent experiments and the bars represent SD between the experiments.

PBMC isolated from HLA-A*02:01-positive, HSV-1 seropositive SYMP and ASYMP individuals was stained for co-stimulatory and survival molecules to further evaluate any defect in the homeostatic maintenance of gB_183–191_-specific CD8^+^ T cells in SYMP vs. ASYMP individuals. We studied the surface expression of co-stimulatory molecule CD28 and survival molecule CD127 on PBMC isolated from HSV-seropositive SYMP and ASYMP individuals (Figure 5). Figure 5A shows a representative contour plot of CD28 and CD57 expression gated on gB_183–191_ tetramer in PBMC isolated from SYMP and ASYMP individuals. The frequency of the CD28^+^gB_183–191_-specific CD8^+^ T cells was significantly (*p* = 0.003) lower in SYMP than in ASYMP individuals. The median frequency of CD28^+^gB_183–191_-specific CD8^+^ T cells in SYMP individuals was 54.1%, whereas ASYMP individuals showed 25.6% (Figure 5B). Furthermore, we counted the CD28^+^gB_183–191_-specific CD8^+^ T cells and observed a significant (*p* = 0.02%) difference in the absolute number between SYMP and ASYMP individuals. The low expression of co-stimulatory molecules was further confirmed by reduced proliferation in CD8^+^ T cells isolated from SYMP individuals (Figure 5C). Then, we investigated whether there were any defects in the homeostatic maintenance of gB_183–191_-specific CD8^+^ T cells in SYMP vs. ASYMP individuals. As expected, the expression of survival molecule CD127 was lower on gB_183–191_-specific CD8^+^ T cells isolated from SYMP individuals than from ASYMP individuals. Figure 5D shows representative histograms for the expression level of survival molecule CD127 gated on gB_183–191_-specific CD8^+^ T cells. The frequency of CD127 gated on gB_183–191_-specific CD8^+^ T cells was significantly (*p* = 0.02) lower in SYMP than in ASYMP individuals (Figure 5E). The median frequency of CD127^+^gB_183–191_-specific CD8^+^ T cells was recorded as 90.1% in ASYMP individuals, whereas SYMP individuals showed 74.9%. Similarly, a significant (*p* = 0.04) difference in the absolute number of CD127^+^gB_183–191_-specific CD8^+^ T cells was observed. The lower absolute number of gB_183–191_-specific CD8^+^ T cells expressing the survival molecule was recorded in SYMP individuals compared to ASYMP individuals (Figure 5F). No significant difference in the Mean Fluorescent Intensity (MFI) of CD127 expression on gB_183–191_-specific CD8^+^ T cells was observed between SYMP and ASYMP individuals (Figure 5G). Open circles indicate data for ASYMP individuals and filled circles represent data for SYMP individuals. The results are representative of two independent experiments for each individual.

### 3.5. Decreased Production of Effector Molecules Granzyme B and Perforin by HSV-Specific CD8^+^ T Cells Isolated from HSV-1 Seropositive SYMP Individuals

**Figure 6:**
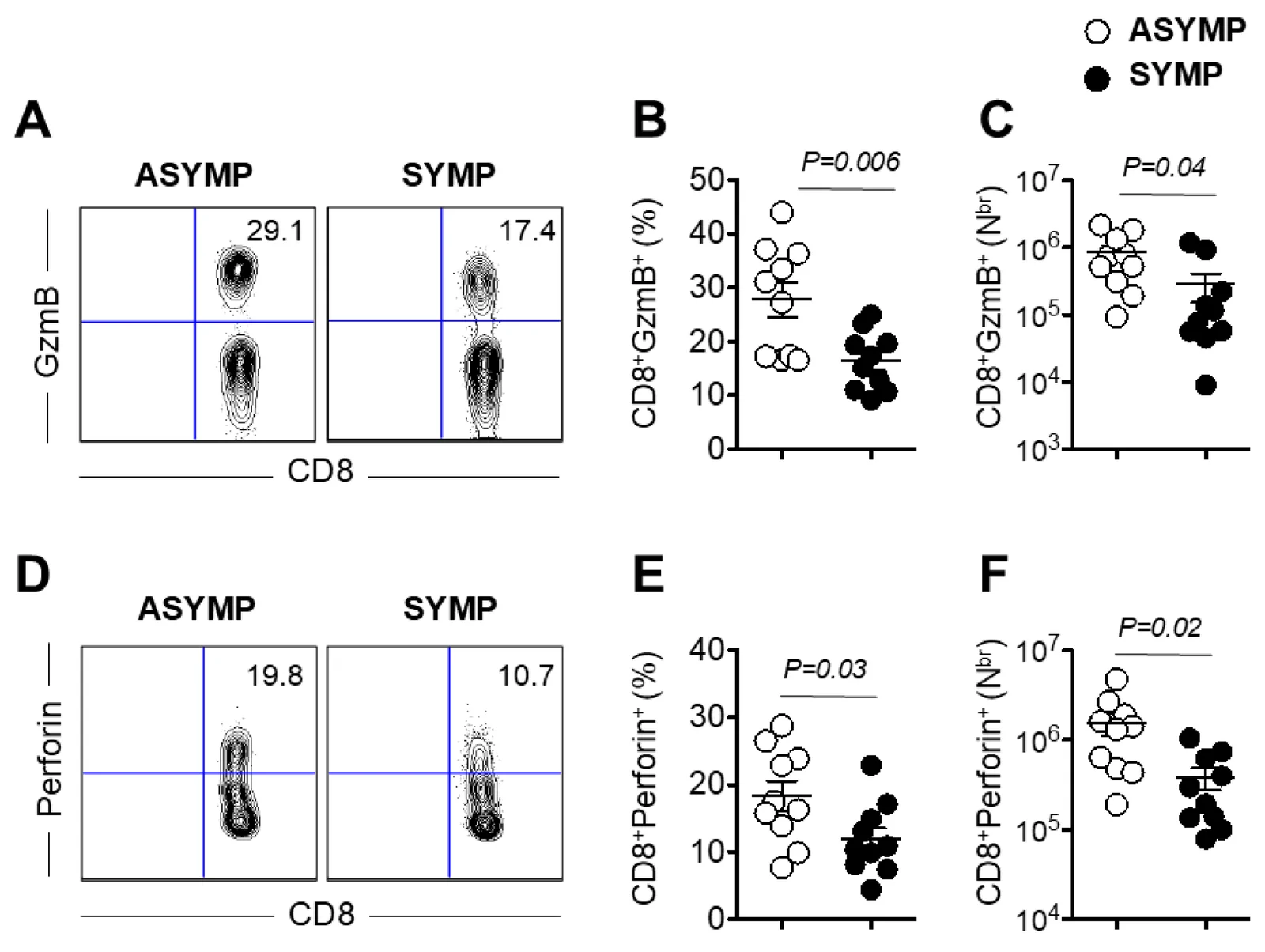
Decreased production of effector molecules Granzyme B and Perforin by HSV-specific CD8^+^ T cells isolated from HSV-1 seropositive SYMP individuals: PBMC isolated from HLA-A*02:01-positive, HSV-1 seropositive SYMP and ASYMP individuals were stimulated and then stained for effector molecules granzyme B and perforin (**A**). Representative contour plot of CD8^+^GzmB^+^ cells gated on gB_183–191_-specific CD8^+^ T cells in PBMC isolated from SYMP and ASYMP individuals. (**B**) The frequency of CD8^+^GzmB^+^ gated on gB_183–191_-specific CD8^+^ T cells in SYMP and ASYMP individuals (**C**). The absolute number of CD8^+^GzmB^+^ gated on gB_183–191_-specific CD8^+^ T cells in SYMP and ASYMP individuals. (**D**) The contour plot of CD8^+^Perforin^+^ cells gated on gB_183–191_-specific CD8^+^ T cells in PBMC isolated from SYMP and ASYMP individuals. (**E**). Frequency of CD8^+^Perforin^+^ gated on gB_183–191_-specific CD8^+^ T cells in SYMP and ASYMP individuals. (**F**). The absolute number of CD8^+^Perforin^+^ gated on gB_183–191_-specific CD8^+^ T cells in SYMP and ASYMP individuals. Open circles indicate data for ASYMP individuals and filled circles represent data for SYMP individuals. The results are representative of two independent experiments for each individual.

PBMC isolated from HLA-A*02:01-positive, HSV-1 seropositive SYMP and ASYMP individuals was stimulated and then stained for effector molecules granzyme B and perforin (Figure 6). Figure 6B shows a representative contour plot of CD8^+^GzmB^+^ cells gated on gB_183–191_-specific CD8^+^ T cells in PBMC isolated from SYMP and ASYMP individuals. We observed a significant difference (*p* = 0.006) in the frequency of CD8^+^GzmB^+^ gated on gB_183–191_-specific CD8^+^ T cells from SYMP as compared to ASYMP individuals (Figure 6B). The median frequency of CD8^+^GzmB^+^ gated on gB_183–191_-specific CD8^+^ T cells in SYMP individuals was recorded as 17.4%, whereas ASYMP individuals showed 29.1%. We also observed a significant (*p* = 0.03) difference in the absolute number of CD8^+^GzmB^+^ gated on gB_183–191_-specific CD8^+^ T cells. ASYMP individuals harbored a higher absolute number of CD8^+^GzmB^+^ gated on gB_183–191_-specific CD8^+^ T cells compared to SYMP individuals (Figure 6C). Similarly, the expression of another effector molecule, Perforin, was evaluated on HSV-specific CD8^+^ T cells. Figure 6D shows a contour plot of CD8^+^Perforin^+^ cells gated on gB_183–191_-specific CD8^+^ T cells in PBMC isolated from SYMP and ASYMP individuals. A significant difference (*p* = 0.03) was observed in the frequency of CD8^+^Perforin^+^ gated on gB_183–191_-specific CD8^+^ T cells isolated from SYMP as compared to ASYMP individuals (Figure 6E). The median frequency of CD8^+^Perforin^+^ gated on gB_183–191_-specific CD8^+^ T cells in the SYMP individual was recorded as 10.7%, whereas in ASYMP, it was 19.8%. We also observed a significant (*p* = 0.02) difference in the absolute number of CD8^+^Perforin^+^ gated on gB_183–191_-specific CD8^+^ T cells. ASYMP individuals presented a higher absolute number of CD8^+^Perforin^+^ gated on gB_183–191_-specific CD8^+^ T cells compared to SYMP individuals (Figure 6F). Open circles indicate data for ASYMP individuals and filled circles represent data for SYMP individuals. The results are representative of two independent experiments for each individual. Altogether, these results indicated a significantly higher frequency of HSV-specific senescent CD8^+^ T cells in SYMP individuals, which were exhausted, lacked proliferative capacity, showed decreased expression of a co-stimulatory molecule, were less functional, and could not maintain homeostatic proliferation as compared to HSV-seropositive ASYMP individuals.

## 4. Discussion

Immunosenescence is marked by a progressive increase in the number of memory CD8^+^ T cells that exhibit poor functional responsiveness to infections and an increase in autoimmune disorders and cancers [26,27]. In this report, we propose that immunosenescence may contribute to dysfunctional CD8^+^ T cell cytotoxicity against the reactivating HSV-1 virus in SYMP individuals, whereas CD8^+^ T cells remain intact in ASYMP individuals. Another key question we address is whether there are differences in the maintenance of an optimal CD8^+^ T cell response between HSV-1 seropositive ASYMP and SYMP individuals.

Senescent CD8^+^ T cells retain a limited degree of responsiveness to antigenic stimulation despite their defective replication abilities. These CD8^+^ T cells express higher levels of T cell activation markers [28], senescence, and immune exhaustion markers PD-1, KLRG1, and CD57 [29] but are deficient in co-stimulation [30] and T-cell survival markers. Terminally differentiated or senescent CD8^+^ T cells display a distinct phenotype characterized by downregulation of the costimulatory molecules CD28 and/or CD27, upregulation of natural killer cell-like receptors such as killer-cell lectin-like receptor G1 (KLRG1), and elevated expression of CD57, a primary marker of senescence [31]. Despite impaired proliferative ability, these cells acquire cytotoxic functions, producing granzyme B and perforin [32]. The turnover rate of CD57^+^CD8^+^ T cells has been shown to increase in cases of immune dysregulation and autoimmune diseases [16]. Several chronic viral infections such as HIV, HCMV, EBV, hepatitis C virus, human parvovirus B_19_, Kaposi sarcoma as well as natural acute measles infection in children show increased populations of CD8^+^CD28^−^ (CD8^+^CD57^+^) T-cells (Reviewed in [33,34]). In HIV-infected individuals, there is an increased frequency of CD8⁺CD28^−^CD57⁺ T cells, which correlates with disease progression. Similarly, in individuals infected with HCMV, there is a significant increase in CD8⁺CD28⁻CD57⁺ T cells, which are often oligoclonal and antigen specific. These cells are thought to play a role in immunoregulation and may influence disease outcomes. The expansion of CD8⁺CD28^−^CD57⁺ T cells has also been observed in EBV infections, suggesting their involvement in the immune response to chronic viral antigens. In chronic HCV infection, the accumulation of CD8⁺CD28^−^ T cells correlates with liver pathology, highlighting their potential role in disease progression. Likewise, Kaposi sarcoma patients show a significant increase in the frequency of CD8⁺CD28^−^CD57⁺ T cells, suggesting their involvement in the pathogenesis of this malignancy. In concordance with studies on several viral infections, our findings indicate a similar phenomenon. We demonstrated that HSV-specific CD8^+^ T cells from SYMP individuals expressed increased levels of CD57 along with decreased expression of CD28. Additionally, the decreased proportion of CD28^+^CD8^+^T cells in SYMP individuals also presents further evidence for immunosenescence. Chronic HCV infection causes a contraction in early-differentiated CD28^+^ HCV and HIV-specific T cells, which indicates that virus-infected cytotoxic T cells reach a state of replicative senescence [13,20,35,36]. Increased CD8^+^CD57^+^ T-cell populations were also reported in the peripheral blood of patients with pulmonary tuberculosis [37]. Recent reports on SARS-CoV-2 infection in non-elderly patients showed expansion of effector CD8^+^ T cells producing cytotoxic molecules such as granzymes A and B and perforin, along with increased PD-1 expression on CD8 T cells [38,39,40]. The oligoclonal expansion of HSV-1 specific CD8^+^CD57^+^ T cells is associated with the increased recurrence of infections, similar to other chronic viral infections and cancers. Moreover, the high expression of perforin and Granzyme B observed in ASYMP HSV-1 individuals suggests antiviral and protective immune responses.

Immune activation in HIV-infected individuals demonstrates significantly decreased CD127 expression and increased CD57 expression, indicating a T cell subset with limited renewal capacity and reduced survival competency [41]. Previous studies on chronic HIV infections have shown that the progressive loss of CD127 leads to increased apoptosis of CD8^+^ T cells [42]. Our data also suggest that HSV infection in SYMP individuals enhances the differentiation of CD8^+^ T cells toward a late-differentiation phenotype, which could be defective in virus elimination. We observed a lower expression of CD127 on HSV-specific CD8^+^ T cells from SYMP individuals compared to ASYMP individuals. CD57 and KLRG1 expression increase with age and correlate with effector memory subsets. Several reports have shown an association of CD57 and KLRG1 with the impaired function of CD8^+^ T cells [16,43,44]. The T-box transcription factor T-bet plays a crucial role in determining the differential fate of CD8^+^ T cells responding to infection, optimal memory, and terminal differentiation. In CD8^+^ T cells, T-bet is upregulated upon activation and is associated with the induction of effector functions [45]. Increased expression of CD57, along with a high levels of KLRG1 and low levels of CD28, are the distinguishing characteristics of senescent CD8^+^ T cells [21]. Our data demonstrated a significantly higher frequency of HSV-specific CD57^+^CD8^+^ T cells, KLRG-1^+^CD57^+^CD8^+^ T cells, and T-bet^+^CD57^+^CD8^+^ T cells in SYMP individuals compared to ASYMP individuals. The expression of T-bet was increased in CD8^+^ T cells from SYMP individuals and correlated with CD57 and KLRG1 expression. It was also shown that CD57 expression strongly correlates with the simultaneous expression of granzymes and perforin in CD8^+^ T cells. Although senescent CD8^+^ T cells typically have preserved effector function (i.e., the ability to produce cytokines and even kill target cells), a lack of proliferative potential impairs their ability to mount robust immune responses and expand in number upon reactivation [46,47]. The relationship between T cell senescence and HSV recurrence is likely complex and influenced by various factors, including the frequency and severity of HSV infections. While frequent HSV recurrences can contribute to T cell senescence, senescent T cells may also worsen symptoms of HSV recurrence by impairing immune responses. We have previously shown that in LAT (−) deficient HSV-mutant, which has reduced reactivation ability, CD8^+^ T-cells are fully functional and control the HSV infection, in contrast to LAT(+) HSV wild type with higher reactivation frequency and more dysfunctional CD8^+^ T-cells [48,49].

**Figure 7:**
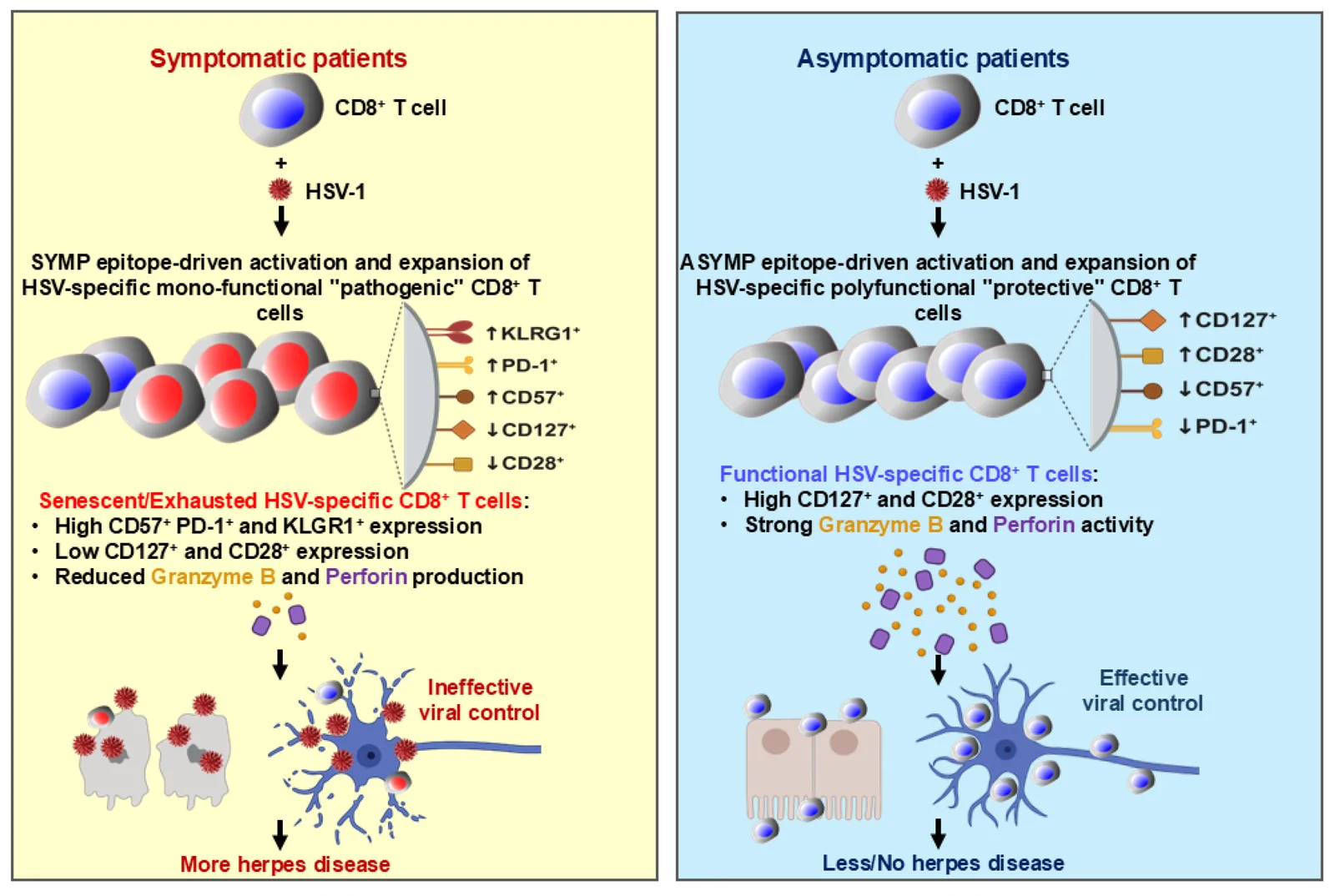
Illustration of the mechanisms of senescent and exhausted HSV-specific CD8^+^ T cells in SYMP individuals. Symptomatic patients (**left**) exhibited senescent and exhausted HSV-specific CD8^+^ T cells. While the majority of the cells exhibited a senescent profile, characterized by high CD57^+^ PD-1^+^ and KLGR1^+^ expression, low CD127^+^ and CD28^+^, and reduced cytotoxic function, a small fraction of newly recruited cells exhibited functional profiles. This major dysfunction led to more frequent HSV-1 reactivation and increased viral spread, resulting in greater cell damage and disease severity. In contrast, Asymptomatic patients (**right**) show high numbers of functional HSV-specific CD8^+^ T cells expressing CD127^+^, CD28^+^, Granzyme B+, and Perforin+, effectively controlling virus reactivation and limiting disease progression.

In this report, we have demonstrated that intermittent HSV-1 reactivation in SYMP individuals leads to the loss of early-differentiated CD8^+^ T cells and progressive accumulation of frequently activated, late-differentiated senescent CD8^+^ T cells. We conclude that immunological changes in CD8^+^ T cells, as well as increased expression of CD57, PD-1, KLRG1, and T-bet from SYMP individuals (as illustrated in Figure 7), eventually drive them toward the end stage of senescence, potentially favoring viral persistence. In contrast, ASYMP individuals retain functional CD8^+^ T cells characterized by higher CD28, CD127, perforin, and granzyme 20B expression, suggesting anti-viral and protective immune responses. It is possible that a senescent population of CD8^+^ T cells in SYMP individuals contributes to maintaining latency, while another, more active CD8^+^ T cell population is recruited to respond to recurrence when needed, exhibiting increased granzyme and perforin expression. However, further investigation is required to evaluate whether a distinct population of CD8^+^ T cells with heightened cytotoxic function is recruited during reactivation.

Our findings suggest a significant correlation between T cell senescence and HSV recurrence; however, the causal relationship remains unclear. Future mechanistic studies are needed to better delineate this relationship. Also, this study focused on individuals expressing HLA-A*02:01, with analyses conducted exclusively on a specific gB peptide (183–191). While this approach enabled a detailed assessment of antigen-specific immune responses within this cohort, it did not fully capture the diversity of HLA alleles in the global population. Future studies should investigate additional HLA-restricted epitopes to evaluate immune responses across a broader range of individuals.

Thus, the present study highlights the critical role of CD8^+^ T cell senescence in HSV-1 pathogenesis, emphasizing the need to develop immunotherapeutic approaches to restore functional CD8^+^ T cell and control recurrent Herpes-related infections.

## Figures and Tables

**Table 1:** Cohorts of HLA-A*02:01 positive, HSV seropositive Symptomatic and Asymptomatic individuals enrolled in the study.

Subject-Level Characteristic	All Subjects (*n* = 781)
Gender [no. (%)]:	
Female	395 (51%)
Male	386 (49%)
Race [no. (%)]:	
White	543 (69%)
Nonwhite	238 (31%)
Age [median (range) year]:	30 (21–67 year)
HSV status [no. (%)]	
HSV-1-positive	283 (36%)
HSV-2-positive	366 (47%)
HSV-1-& 2-positive	31 (4%)
HSV-negative	92 (12%)
HLA [no. (%)]	
HLA-A*02:01-positive	411 (53%)
HLA-A*02:01-negative	370 (447%)
Herpes Disease Status [no. (%)]	
ASYMPTOMATIC (ASYMP)	711 (91%)
SYMPTOMATIC (SYMP)	70 (9%)

## Data Availability

The original contributions presented in this study are included in the article/Supplementary Material. Further inquiries can be directed to the corresponding author.

## Supplementary Materials

The following supporting information can be downloaded at: https://www.mdpi.com/article/10.3390/v17050606/s1, Figure S1: Example of flow cytometry gating strategy for PD-1^+^ HSV-1-specific CD8^+^ T cells: PBMC was stained with a panel of antibodies. The cell population was first plotted on an ungated plot showing size and granularity (A). Then, a first gate (R1) was performed on lymphocyte area and plotted as single cells (B). Next, a second gate (R2) was performed and the cell population was re-plotted based on CD8 and gB_183–191_ tetramers (C). Finally, a third gate (R3) was performed on CD8^+^ gB_183–191_ tetramers + T cells and plotted on overly histogram of PD-1 expression (D).
