# Supplementary material for: Dysfunctional Senescent Herpes Simplex Virus-Specific CD57+CD8+ T Cells Are Associated with Symptomatic Recurrent Ocular Herpes in Humans
Source: Viruses. 2025 Apr 24;17(5):606. doi: 10.3390/v17050606 (PMC12115701; doi:10.3390/v17050606)

Supplementary Materials

Figure S1. Example of flow cytometry gating strategy for PD-1+ HSV-1-specific CD8+ T cells: PBMC was stained with a panel of antibodies. The cell population was first plotted on an ungated plot showing size and granularity (A). Then, a first gate (R1) was performed on lymphocyte area and plotted as single cells (B). Next, a second gate (R2) was performed and the cell population was re-plotted based on CD8 and gB183–191 tetramers (C). Finally, a third gate (R3) was performed on CD8+ gB183–191 tetramers + T cells and plotted on overly histogram of PD-1 expression (D).

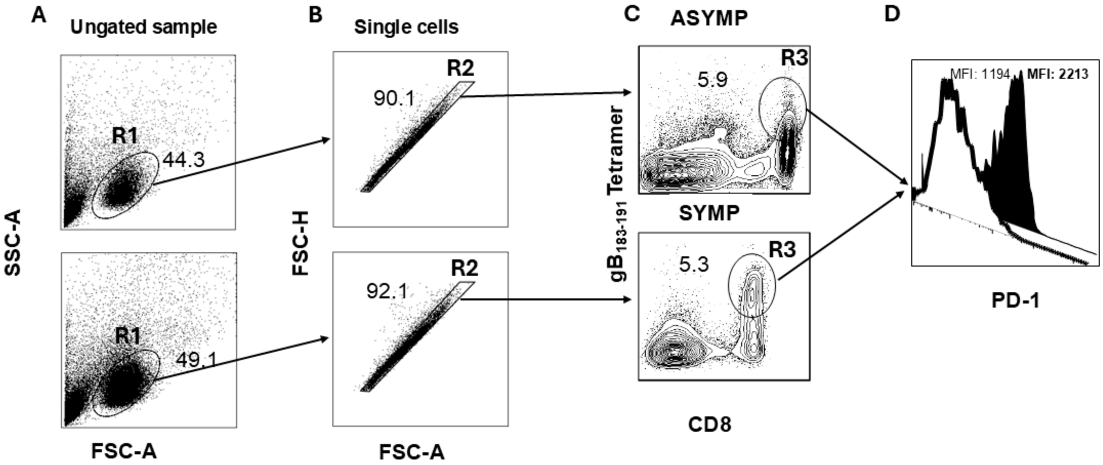

Supplement: Supplementary file 1 [file viruses-17-00606-s001.zip › viruses-3525481-supplementary.pdf]
